# Reprogramming viral immune evasion for a rational design of next-generation vaccines for RNA viruses

**DOI:** 10.3389/fimmu.2023.1172000

**Published:** 2023-04-17

**Authors:** Chia-Ming Su, Yijun Du, Raymond R. R. Rowland, Qiuhong Wang, Dongwan Yoo

**Affiliations:** ^1^ Department of Pathobiology, College of Veterinary Medicine, University of Illinois at Urbana-Champaign, Urbana, IL, United States; ^2^ Shandong Key Laboratory of Animal Disease Control and Breeding, Institute of Animal Science and Veterinary Medicine, Shandong Academy of Agricultural Sciences, Jinan, Shandong, China; ^3^ Center for Food Animal Health, Department of Animal Sciences, College of Food, Agricultural, and Environmental Sciences, The Ohio State University, Wooster, OH, United States; ^4^ Department of Veterinary Preventive Medicine, College of Veterinary Medicine, The Ohio State University, Columbus, OH, United States

**Keywords:** type I interferons (IFNs), NF-kappa B (NF-κB), IFN antagonism, live-attenuated vaccine, veterinary vaccine, viral immune evasion, next-generation vaccines, veterinary virology

## Abstract

Type I interferons (IFNs-α/β) are antiviral cytokines that constitute the innate immunity of hosts to fight against viral infections. Recent studies, however, have revealed the pleiotropic functions of IFNs, in addition to their antiviral activities, for the priming of activation and maturation of adaptive immunity. In turn, many viruses have developed various strategies to counteract the IFN response and to evade the host immune system for their benefits. The inefficient innate immunity and delayed adaptive response fail to clear of invading viruses and negatively affect the efficacy of vaccines. A better understanding of evasion strategies will provide opportunities to revert the viral IFN antagonism. Furthermore, IFN antagonism-deficient viruses can be generated by reverse genetics technology. Such viruses can potentially serve as next-generation vaccines that can induce effective and broad-spectrum responses for both innate and adaptive immunities for various pathogens. This review describes the recent advances in developing IFN antagonism-deficient viruses, their immune evasion and attenuated phenotypes in natural host animal species, and future potential as veterinary vaccines.

## 1 Introduction

Virus-host interactions play a vital role during infection and determine pathogenic consequences, including host and tissue tropisms, viral elimination and persistence, tumorigenesis, and clinical outcomes. Physical and chemical barriers, innate immune systems, and various types of immune cells are considered the first line of defense of a host against viral infections. Innate and adaptive immunities are two main surveillance systems to resolve viral infections. Immediately upon infection, the type I interferon (IFNs-α/β) production system is activated by recognizing viral components by host cell factors. It is the most effective cellular defense against viral infection in the early stage of infection (1). In turn, viruses have developed distinct immune-disarming abilities to facilitate replication. The modulation of IFN functions is an effective strategy for invading viruses to survive in the host (2). IFN suppression by viruses can also negatively impact the immunogenicity of live-attenuated vaccines (3). With the advances in the study of viral immune evasion mechanisms and reverse genetics technology, it is now possible to remove the IFN suppression function from target viruses and design new generation vaccines for diverse RNA viruses. Indeed, some engineered viruses have been constructed and examined in their natural host animal species for immunogenicity and clinical outcomes (4, 5). This article discusses the molecular mechanisms for viral IFN antagonism, generation of IFN antagonism-deficient viruses, their immunogenic consequences in animals following vaccination, and prospects as next-generation vaccines for veterinary diseases.

## 2 Innate immune response to viral infection

### 2.1 Type I interferons and innate immunity

Immune cells involved in the innate immune system represent monocytes, macrophages, eosinophils, neutrophils, and natural killer (NK) cells. IFNs-α/β are antiviral cytokines and constitute one of the most critical components in the innate immune system against invading viruses. The IFNs-α/β signaling triggers expression of a series of IFN-stimulated genes (ISGs) and contributes to the antiviral state of the cell (6). All nucleated cells can produce IFNs-α/β, but plasmacytoid dendritic cells (pDC) are the main type that produce type I IFNs most abundantly (7).

Once an RNA virus enters the cell, viral components trigger a series of intricate recognition mechanisms within the cell (8). The innate immune signaling cascade starts with the recognition of pathogen-associated molecular patterns (PAMPs) by pattern recognition receptors (PRRs) that are composed of fours groups of cellular proteins; nucleotide-binding oligomerization domain (NOD)-like receptors (NLRs), retinoic acid-inducible gene I (RIG-I)-like receptors (RLRs), C-type lectin receptors (CLRs), and Toll-like receptors (TLRs). NLRs, RLRs, and CLRs reside in the cytoplasm, whereas TLRs locate in the endosomal membrane (9). The viral RNA binds to RIG-I or melanoma differentiation-associated gene 5 (MDA5) and triggers conformational changes to expose its caspase activation and recruitment domain (CARD) at the N-terminus. CARD-CARD dimers are then formed and recruit the IFN-β promoter stimulator-1 (IPS-1) [also known as CARD adaptor inducing IFN-β (Cardif), mitochondrial antiviral-signaling protein (MAVS), or virus-induced signaling adaptor (VISA)] (10, 11). IPS-1 recruits the NF-kappa-B essential modulator (NEMO), tumor necrosis factor receptor-associated factor (TRAF) 3, and TRAF family-member-associated NF-κB activator (TANK) proteins, and its complex activates TANK binding kinase 1 (TBK1) and inhibitor of kappa B (IκB) kinase ϵ (IKKϵ). Subsequently, IFN regulatory factor 3 (IRF3) and IRF7 are phosphorylated in the TBK1- and IKKϵ-dependent manners (12). Then, phosphorylated IRF3 and IRF7 form a homodimer or heterodimer for translocation to the nucleus (13), where the IRF3- or IRF7-dimer forms an enhanceosome which binds to the positive regulatory domains (PRDs) I-III regions in the IFN promoters for IFN gene transcriptions (14, 15). IPS-1 may also activate IKKα and IKKβ to trigger the degradation of IkB to free up NF-κB. The released NF-κB binds to PRDs of respective IFN genes and proinflammatory cytokine promoters (15–17). Besides the RIG-I-mediated signaling cascades, TLR3 and TLR7 also recognize double-stranded RNA and single-stranded RNA, respectively, in the endosome (18). TLR3 can recruit the TRIF, nucleosome assembly protein 1 (NAP1), and TRAF3 adaptors and activates IRF3 for IFN expression and downstream signaling for many antiviral proteins (9, 15).

Once IFNs are produced, they are secreted from the cell and bind to their receptors, interferon alpha and beta receptor subunit 1 (IFNAR1) and IFNAR2, on the surface of the same cell (autocrine) or adjacent cells (paracrine). The binding of IFNs to their receptors triggers the Janus kinase (JAK)-signal transducers and activator of transcription (STAT) signaling pathway. The activation of JAK1 and tyrosine kinase 2 (Tyk2) is the first response to the IFN signaling and results in phosphorylation and dimerization of STAT1 and STAT2, followed by the recruitment of IRF9 to form the IFN-stimulated gene factor 3 (ISGF3) complex. The ISGF3 complex then translocates to the nucleus. It induces the expression of ISGs by binding to the IFN-stimulated regulatory response elements (ISRE) in the promoters of ISGs (19). More than 300 ISGs have been identified so far, and they are the major executors of IFNs acting as effector molecules for the antiviral response (19, 20).

### 2.2 Pleiotropic role of type I IFNs and activation of adaptive immunity

In addition to establishing an antiviral state, type I IFNs can enhance adaptive immunity by targeting dendritic cells (DCs), NK cells, T cells, and B cells (19). For DCs, type I IFNs play a key role in the generation and function of DCs (21), suggesting that type I IFNs represent a potent natural adjuvant for crossing the innate and adaptive immune systems. Type I IFNs enhance the ability of DCs to cross-present antigens to T cells and modulate antigen survival and processing (22, 23). IFNs regulate the DCs antigen presentation in an autocrine manner (24). Besides DCs, type I IFNs can promote the activation and survival of NK cells directly or indirectly *via* IL-15 *cis* and *trans* presentation (25) and regulate T cells directly through IFNARs on the surface of T cells. Type I IFNs are critical mediators for the spontaneous priming of antitumor CD8+ T cell response (26). In the herpesvirus-infected mouse model, type I IFNs have been shown to stimulate CD4+ T cells for undergoing clonal expansion (27). The T cells primed by type I IFNs also show an increased ability to help B cells to enhance antibody secretion (28). The studies using lymphocytic choriomeningitis virus or West Nile virus as models have also demonstrated that type I IFNs upregulate the survival, maturation, cytotoxicity, and clonal expansion of CD8+ T cells during infection (29–34). In addition to effector CD8+ T cells, memory CD8+ T cells are also regulated by type I IFNs. Studies using vaccinia virus, vesicular stomatitis virus, and lymphocytic choriomeningitis virus have shown that type I IFNs promote the differentiation of memory CD8+ T cells by affecting the initial clonal expansion (31, 35). Furthermore, type I IFNs regulate B cell activation, antibody secretion, and isotype switching during the vesicular stomatitis virus and West Nile virus infections (36–38). By increasing the level of B-cell survival factors, type I IFNs can promote survival and activation of B cells and enhance autoantibody production (39). Thus, it is evident that the immunological functions of type I IFNs are much broader than establishing an antiviral state and play an important role in regulating adaptive immunity.

### 2.3 NF-κB signaling and cytokine responses to viral infection

Nuclear factor-κB (NF-κB) is a family of transcription factors and a key mediator for proinflammatory cytokine production. NF-κB consists of RelA (p65) and RelB, NF-κB1 (p50 and its precursor p105), NF-κB2 (p52 and its precursor p100), and c-Rel to form a homodimer or heterodimer with RelA or RelB. Numerous factors activate the NF-κB signaling including TLR ligands and cytokines such as tumor necrosis factor-α (TNF-α). Upon stimulation, upstream kinases IKKα and IKKβ are activated by phosphorylation. IKKβ then phosphorylates the IκB and degrades IκB through a proteasome-dependent manner. IκB is the negative regulator for NF-κB, and so the IκB degradation releases NF-κB, which is then phosphorylated. The activated NF-κB enters the nucleus and binds to the specific DNA locus, the κB site (40). NF-κB signaling triggers the expression of type I IFNs and various proinflammatory cytokines, including IL-1β, IL-6, IL-8, IL-15, and TNF-α (41). The expressed proinflammatory cytokines prompt the positive feedback loop to the NF-κB signaling in an autocrine manner (42). The protein inhibitor of activated STAT 1 (PIAS1) is the NF-κB negative regulator residing in the nucleus (43). PIAS1 prevents NF-κB dimers from binding to κB sites by binding to p65 in the nucleus (43). For respiratory viral infections, NF-κB is strongly activated in the lungs and results in the induction of proinflammatory cytokines and chemokines (44, 45).

## 3 Viral IFN antagonists and their mode of action

**Figure 1:**
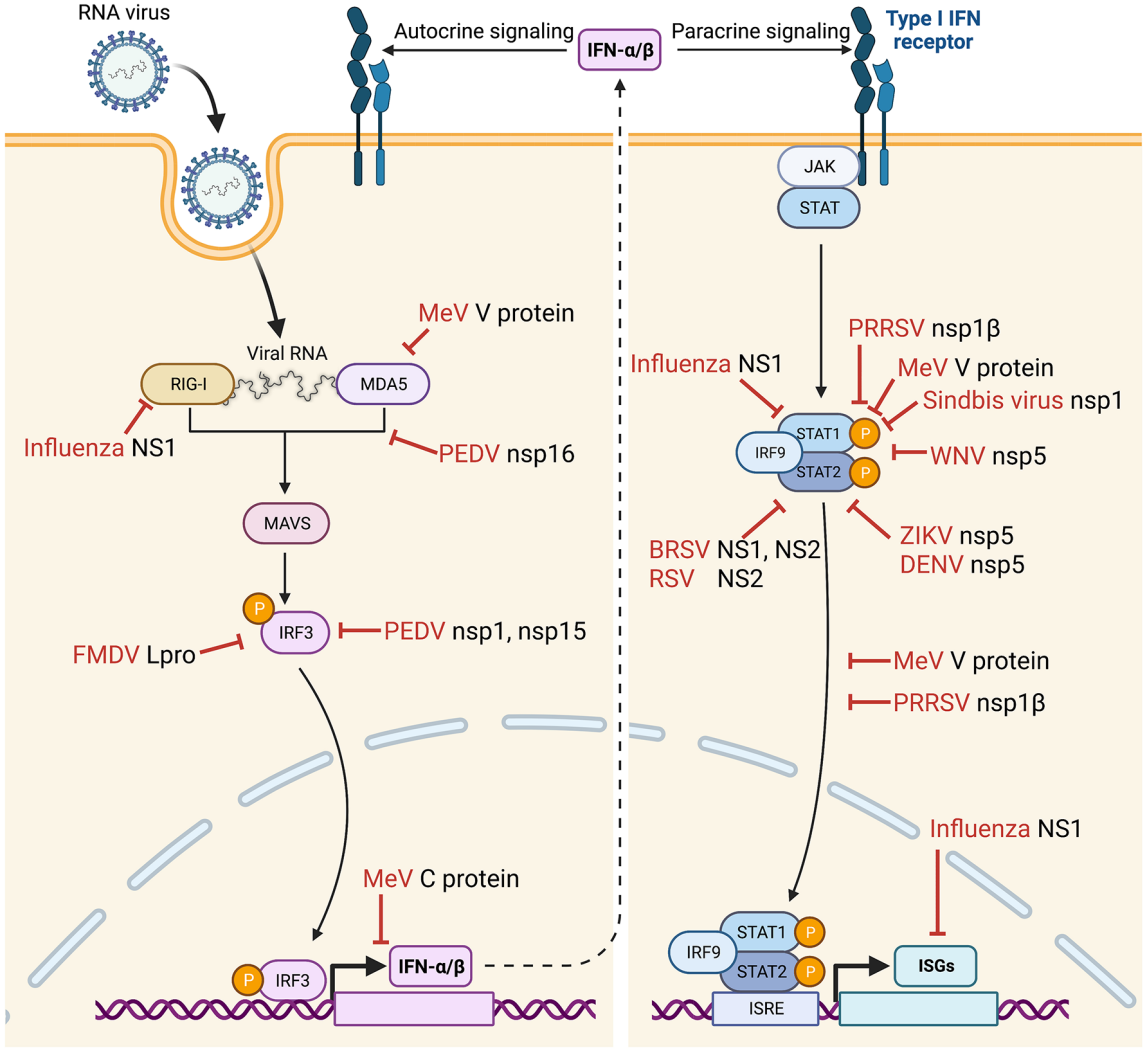
Suppression of IFNs-α/β production and IFN-stimulated gene (ISG) expression during infection by various animal viruses. IFN suppression-deficient viruses have been constructed, and their immunological and clinical outcomes have been examined in their natural host animal species. Vertical arrow (dotted line) divides the IFN signaling to “IFN production pathway” (left) and “IFN signaling JAK-STAT pathway” (right). Bars (red) indicate sites of action by specific viral proteins for IFN suppression. FMDV, foot-and-mouth disease virus (48–51); PEDV, porcine epidemic diarrhea virus (52); MeV, measles virus (53–59); PRRSV, porcine reproductive and respiratory syndrome virus (60–63); WNV, West Nile virus (64); ZIKV, Zika virus (65); DENV, Dengue virus (66); BRSV, bovine respiratory syncytial virus (67–70); RSV, respiratory syncytial virus (71, 72); IRF, interferon regulatory factor; ISRE, interferon stimulation response element; STAT, signal transducer and activator of transcription. Images were created with BioRender.com.

Many viruses code for viral proteins to antagonize the IFN pathway and often produce more than one antagonist (6, 46, 47). Viruses have evolved to employ diverse strategies to evade the IFN system, and some of the viral strategies and mechanisms of action are discussed below (**Figure 1**). For convenience, the signaling cascade for production of type I IFNs in virus-infected cells will be referred to as ‘IFN production pathway’, and the signaling mediated by IFNs for production of ISGs will be referred to as ‘JAK-STAT signaling pathway’.

### 3.1 Inhibition of IFN production pathway

Some viruses suppress the IFN production pathway by disrupting IFN receptor functions, such as reducing receptor expression and degrading receptor molecules. For example, the influenza A virus disrupts IFN signaling by downregulating the expression of IFN receptor. The NS1 protein is a non-structural component encoded by influenza viruses A, B, and C. It counteracts the host antiviral responses by two primary mechanisms: inhibition of the activation of IRF3 and IFN transcription and inhibition of the processing of IFN pre-mRNAs (73). The NS1 protein binds the host cell RNA and forms complexes with RIG-I to antagonize IFN production (74). The NS1 protein also suppresses the expression of both chains of IFNAR receptor at the transcription level (75, 76). Epstein-Barr virus (EBV) establishes a latent infection in B lymphocytes. EBV expresses two isoforms of latent membrane protein 2 (LMP2). Of the two forms, LMP2A plays an essential role in viral latency and progression of EBV-related diseases such as Burkitt’s lymphoma, nasopharyngeal carcinoma, and Hodgkin’s lymphoma. The LMP2A and LMP2B proteins degrade IFN receptors and modulate both type I and type II IFN responses (77).

Activation of IRF3 is an essential step for downstream IFN production signaling (9, 15), and some viruses have developed mechanisms to manipulate the IRF3 activation. Hepatitis C virus NS3-4A is the viral serine protease and has a crucial role in the viral replication cycle. NS3-4A cleaves the polyprotein at four sequential sites to yield NS4A, NS4B, NS5A, and NS5B proteins. NS3-4A can cleave and inactivate the host proteins TRIF (TIR-domain-containing adapter-inducing interferon-β) and Cardif, which are essential adaptors in response to IFNs mediated by TLR3 and RIG-I, respectively. NS3/4A also cleaves IKK and inactivates IRF3 (78). Classical swine fever virus and bovine viral diarrhea virus are member viruses of the Pestivirus genus of the Flaviviridae family. For pestiviruses, N^pro^ is the N-terminal viral protease of the polyprotein and can degrade IRF3 for inactivation (79–81). Besides the direct degradation, IRF3 function may be inhibited by some other viruses. The rabies virus P protein and the hantavirus G1 protein have been determined to suppress IFN signaling by inhibiting IRF3 phosphorylation (82, 83). Rabies virus infects all species warm-blooded animals. It replicates in the cytoplasm of the cell, and the viral RNA is tightly encapsidated by the viral nucleoprotein and the RNA polymerase complex which consists of the phosphoprotein (P). The P protein is a cofactor of RNA polymerase by participating in viral genome transcription and replication. The P protein prevents IFN response in virus-infected cells by targeting TANK-binding kinase-1 (TBK1) and inhibiting phosphorylation and activation of IRF3. Sin Nombre virus, carried by rodents, is an etiologic agent for hantavirus pulmonary syndrome. With a pathogenic strain of hantavirus, the cytoplasmic tail of the G1 glycoprotein was shown to regulate the IFN response by inhibiting IRF3 phosphorylation at the level of the TBK1 complex (84). In contrast, the hantavirus G1-mediated IFN downregulation was absent in the Prospect Hill nonpathogenic strain, indicating that the IFN suppression is an important virulence factor for hantaviruses.

Coronaviruses (CoV) carry diverse strategies to inhibit IRF3 activation. CoV genomes are the largest among all RNA viruses and contain the positive-sense RNA of 25-32 kb in length. The viral genome codes for two large polyproteins, and the nsp1 protein is the N-terminal cleavage product of the polyprotein. The nsp1 protein of SARS-CoV-1 suppresses the IFN response *via* IRF3-dependent downregulation (85, 86), whereas the ORF8b and ORF8ab proteins induce IRF3 degradation *via* the ubiquitin-dependent proteasomal pathway (87). SARS-CoV-2 is an emerging virus with an unknown animal origin. As the causative agent for COVID-19 in humans, it has crossed the species barrier to infect other animal species, including cats, dogs, tigers, lions, mink, white-tail deer, monkeys, ferrets, and hamsters, to name a few (88). Humans can facilitate reverse-zoonotic transmission to animals, and the virus in infected animals may evolve to spill back to humans and other animal species. SARS-CoV-2 codes for nsp3 papain-like protease and nsp5 3C-like protease, and these two viral proteases are responsible for cleaving viral polyproteins for replication. The nsp3 protein cleaves IRF3 directly, and this finding explains the blunted type I IFN response seen in COVID-19 patients during infection (89). Although the nsp12 viral RNA polymerase of SARS-CoV-2 does not impair IRF3 activation, it attenuates type I IFN production by inhibiting IRF3 nuclear translocation (90). Meanwhile, the ORF7a protein decreases IRF3 phosphorylation by downregulating TBK1 expression (91, 92). Similarly, the viral spike (S) protein binds directly to IRF3 and further diminishes its expression, whereas the levels of NF-κB and STAT1 transcription factors remain intact (93). The murine coronavirus mouse hepatitis virus can also delay ISG production by limiting the IRF3 transcription (94).

Besides the direct regulation of IRF3, some viruses inhibit signaling adaptors and regulators in the IFN production pathway. For example, the swine arterivirus, porcine reproductive and respiratory syndrome virus (PRRSV), has been found to inhibit the IFN promoter activity and impairs type I IFN production (60). The nsp1α protein of PRRSV downregulates IFN production by degrading the CREB (cyclic AMP response element-binding)-binding protein (CBP) *via* the ubiquitin-dependent proteasome pathway (95–97). PRRSV also suppresses the NF-κB activation and inhibits type I IFN production in the RIG-I dependent manner (98, 99). A recent study showed that nsp1β of PRRSV binds to nucleoporin 62 (Nup62) (100). Nup62 is a major structural component of the nuclear pore complex (NPC) on the nuclear membrane, and NPC functions as a gateway for nucleocytoplasmic trafficking of nuclear proteins and mRNAs. The binding of nsp1β to Nup62 disrupts the NPC integrity, causing the nuclear retention of host mRNAs and inhibiting the host cell protein production, including IFNs, ISGs, and IRF3 (100).

### 3.2 Inhibition of IFN signaling JAK-STAT pathway

IFNs-α/β are secretory cytokines. Once expressed in virus-infected cells and secreted, they bind to their receptors on the same cell or neighbor cells and activate the IFN signaling pathway, namely the JAK-STAT signaling. Some viruses have developed mechanisms to impair the JAK-STAT pathway by targeting STAT1 and STAT2. Paramyxoviruses disturb the STAT signaling by using diverse mechanisms, such as the direct binding of viral protein to STAT to prevent phosphorylation (101). The paramyxovirus V protein is of particular interest since it regulates the JAK-STAT pathway by degrading STAT1 and STAT2 and mediating their nuclear accumulations (53, 102–104). Sendai virus, a paramyxovirus, produces a set of four C proteins by mRNA editing of the P gene and inhibits IFN-induced tyrosine phosphorylation of STATs (101, 105, 106). All four C proteins bind to STAT1, but only the largest form of C induces the mono-ubiquitination of STAT1 and its degradation (106). Dengue virus (DENV) and Zika virus (ZIKV) in the family *Flaviviridae* have shown the ability to reduce the concentration of STATs in the cells (101, 107, 108). NS5 protein of ZIKV induces the degradation of STAT2 *via* the proteasomal pathway (65, 109).

Respiratory syncytial virus (RSV) has diverse mechanisms to inhibit the type 1 IFN response in different cell types (110). RSV degrades STATs in epithelial cells, whereas, in DCs, it inhibits STAT1 and STAT2 phosphorylation and nuclear accumulation (110, 111). Furthermore, RSV induces STAT2 downregulation through ubiquitin-proteasome degradation by the Elongin-Cullin E3 ligase (112, 113). The porcine arterivirus PRRSV nsp1β protein inhibits STAT1 phosphorylation, leading to inhibition of the JAK-STAT signaling pathway, resulting in the suppression of ISG expressions (60, 61). Other viruses can also block the IFN signaling by targeting IRF9. Such mechanisms are usually found in viruses that cause persistent infections and oncogeneses, such as human papillomavirus, porcine bocavirus, and human T-cell leukemia virus (114–116).

Viruses can block the nuclear accumulation of activated STATs and inhibit IFN signaling. For example, the paramyxovirus virus V protein binds to MDA-5 and decreases the IFN promoter activation (117), as well as binding to STAT1 and STAT2 and preventing their nuclear accumulation (118). The P protein of the rabies virus binds to the DNA-binding and coiled-coil domains of STAT1 to influence the IFN-induced transcription and impairs its nuclear translocation (119). The porcine arterivirus nsp1β protein inhibits STAT1 phosphorylation and further affects the nuclear translocation of ISGF3, thus inhibiting the JAK-STAT signaling pathway (60, 61). PRRSV nsp1β also degrades karyopherin-α1, one of the nuclear transporter proteins, and blocks the ISGF3 nuclear translocation to suppress ISG expressions (120).

## 4 Reprograming viral immune evasion and experimental infections in animals

Of various host immune surveillance systems, the innate immune system builds on a series of antiviral responses. Although the IFN inhibitory ability of a virus is not the only determinant for virulence, it is often one of the most critical virulence factors. Type I IFNs can also prime the activation and maturation of adaptive immune responses. Disturbed and delayed host responses against viral infection are attributed to the suppression of type I IFNs, suggesting that viral inhibition of IFN response may cause the unsatisfactory efficacy of certain vaccines. Thus, future vaccines should stimulate both innate and adaptive immune response. A large body of evidence demonstrates the importance of type I IFNs not only for innate immunity and antiviral function but also for the development of adaptive immunity. Murine norovirus infection in mice, of which type I IFN receptors are deficient, transforms an acute infection into a systemically persistent infection (121). Nevertheless, CD8+ T cell and antibody responses are still enhanced during persistent norovirus infection, suggesting that the deficiency of IFNs leads to viral persistence despite enhanced adaptive immunity (121). Many viruses suppressing the IFN response are known to cause insufficient protection upon vaccination, which makes many vaccines less effective (122).

### 4.1 IFNs as vaccine adjuvants

The potential of IFNs as vaccine adjuvants have been studied by many investigators. The results demonstrate that IFNs play a critical role in generating immune responses to vaccines. IFN treatment is the most effective therapy for controlling persistent hepatitis C virus infection (123–125). IFN-β is helpful for cancer vaccines that induce a greater expansion of tumor-specific CD8+ T cells (126). IFNs have been used as adjuvants coupled with veterinary vaccines. Replicating PRRSVs expressing various types of IFN increased the IFN levels in pigs and conferred protection against PRRSV challenges (127, 128). Recombinant porcine IFN (PoIFNα), used as an adjuvant in PRRSV vaccination, induces adaptive immune response in pigs (129). In other studies, PoIFNα, in combination with inactivated swine influenza virus, significantly upregulated the expression of various immunoregulatory cytokines and higher levels of HA-specific antibodies in 6-week-old pigs (130). Indeed, type I IFNs is a potent adjuvant for influenza vaccines to induce an effective humoral response in mice (131, 132). PoIFNα coupled with a foot-and-mouth disease virus (FMDV) protein vaccine generated more robust immunogenicity and complete protection of pigs from virulent challenge (133). In other FMDV studies, IFNα was endogenously expressed in mice using an adenovirus vector, which improved the generation of T helper cells and the production of all classes of IgG immunoglobulins, especially IgG1 and IgG2a subtypes (134, 135). Endogenous IFNα expressed using a plasmid linked to the equine encephalitis virus (EEV) vaccine showed protection after being administered 24 hours before the challenge (136, 137). This result highlights that up to 24 hours is required to develop the IFN-mediated antiviral response. An IFN inducer coupled with a non-replicating vaccine for the rabies virus provided better protection in rhesus monkeys than the vaccine alone (138).

Taken together, such studies provide insights into the potential application of IFNs as an adjuvant for improving vaccine efficacy. Moreover, reprogramming the viral IFN evasion strategy has allowed the conceptual development of new vaccine designs. The approaches have proven useful in enhancing IFN response during vaccination and improving innate and adaptive immunities. Live attenuated vaccines are generally preferred in veterinary medicine and are considered more protective than inactivated vaccines. Live attenuated vaccines elicit both humoral and cellular immunities, whereas inactivated vaccines induce mainly antibody response. Removing IFN antagonism from viruses has been a strategy for developing both human and veterinary vaccines to achieve better immunogenicity and improved protection. In this article, however, we limit our discussions to only viruses that have been examined in the natural host animals, excluding human trials, as potential vaccine candidates against viral infections in animals.

### 4.2 Picornaviridae

#### 4.2.1 Foot-and-mouth disease virus

FMDV is the prototype member of the *Aphthovirus* genus in the *Picornaviridae* family. FMDV is a highly contagious disease of cloven-hoofed animals in many livestock-producing countries worldwide. Despite the importance of the disease, difficulties in inducing and maintaining an adequate immune response in vaccinated animals have been an issue in controlling FMDV infection. FMDV can suppress the type I IFN response and persists in the tonsils of infected animals for up to 2 years (139). Among the viral proteins, the leader protein L^pro^ stands out as the most effective IFN suppressor of FMDV (**Figure 1**) (48–51). L^pro^ of FMDV is also a deubiquitinase (DUB) and deISGylase and interacts with ISG15 to induce its degradation (140). The FMDV mutant with modified deISGylase activity showed viral attenuation in mice (141). Deletion of L^pro^ from FMDV resulted in a slightly slower growth than wild-type virus in cells and was attenuated in mice (142). Therefore, L^pro^ is the target of the attenuated vaccine design for FMDV in natural host animal species. After removing the IFN suppression function from L^pro^, the IFN suppression-negative FMDV induces higher levels of IFNs and ISGs with a robust neutralizing antibody response in vaccinated pigs (143, 144). Furthermore, pigs were completely protected from the high dose challenges of wild-type virus (143). Besides swine, the virulence of FMDV with L^pro^ mutation was investigated in bovine (145). In this study, a 57 nucleotide in-frame insertion was made in the region between two functional AUG initiation codons of L^pro^. Both wild-type and insertional L^pro^ mutant viruses established primary infection in the nasopharyngeal mucosa with subsequent dissemination to the lungs of the cattle. Insertional L^pro^ mutant FMDV, however, replicated slower and showed quantitatively lower viral loads in secretions and infected tissues and reduced clinical disease, indicating the L^pro^ mutant FMDV was attenuated in cattle. In another study, a recombinant FMDV was generated to completely delete the L^pro^ gene. The L^pro^-deficient FMDV provided 100% protection in cattle from challenges with wild-type virus (146), demonstrating the potential use of L^pro^ mutants in developing live attenuated vaccines for FMD.

### 4.3 Togaviridae

#### 4.3.1 Sindbis virus

Alphavirus is the sole genus of the family *Togaviridae*, consisting of a group of positive-sense, single-stranded RNA viruses. Sindbis virus and Ross River virus are prototype viruses in the Alphavirus genus. Sindbis virus is one of the most widely distributed mosquito-borne viruses and is constantly found in insects and vertebrates. Sindbis virus infection causes polyarthritis, rash, and fever, although most infections are asymptomatic. It has been used as a model virus to study the molecular biology of togaviruses. The major virulence factor of the Sindbis virus is nsp1, which takes part in the inhibition of JAK-STAT signaling (**Figure 1**) (147). The neurovirulent strain AR86 inhibits tyrosine phosphorylation of STAT1 and STAT2, whereas two other closely-related strains, Girdwood and TR339, do not cause significant disease in adult mice and inhibit the JAK-STAT signaling pathway relatively inefficiently (147). Further, threonine at position 538 in the nsp1 protein of strain AR86 was identified as required for efficient disruption of STAT1 activation, while introducing threonine at position 538 to the Girdwood strain fully restored the ability to inhibit the JAK-STAT signaling and virulence in mice (147–149). A similar pathogenic effect has been demonstrated for the Ross River virus, a distantly related alphavirus, indicating that nsp1-mediated IFN suppression function can be removed from alphaviruses, and live-attenuated vaccine candidates can be developed (150–152).

### 4.4 Flaviviridae

#### 4.4.1 West Nile virus and Zika virus

Flaviviruses are vector-borne viruses transmitted by arthropods. Flaviviruses cause a tremendous disease burden for humans and animals, causing millions of human infections annually. West Nile virus (WNV) can cause acute encephalitis and high morbidity especially in horses and birds. The flaviviruses suppress host innate immune responses during infection, and the viral protein NS5 functions as an IFN antagonist by inhibiting the JAK-STAT signaling pathway (**Figure 1**) (64). NS5 inhibits IFN-dependent STAT1 phosphorylation or STAT2 degradation (153, 154). Kunjin virus is a naturally attenuated subtype of WNV. The molecular analyses of the Kunjin virus have identified serine 653 in NS5 as a potent amino acid residue that participates in viral attenuation and IFN modulation (154). After changing the amino acid residue S653 of NS5 to F653, the Kunjin virus restored the ability to downregulate JAK-STAT signaling. Similarly, the amino acid change from phenylalanine to serine in NS5 of the virulent strain of WNV resulted in the loss of the ability to inhibit the JAK-STAT signaling (154). In a mouse model, highly virulent WNV and WNV-like African Koutango virus infections produced severe neurological disease and higher morbidity. In addition, the enhanced virulence of WNV was associated with poor viral clearance and poor induction of neutralizing antibodies (155). Analogous to WNV, the virulence of the ZIKV results from the degradation of STAT2 mediated by NS5 (65). However, the IFN degradation by ZIKV NS5 is host species-restricted and functional for human and nonhuman primate but not for mouse (65). Like ZIKV, DENV NS5 does not antagonize IFN signaling in mice since the DENV NS5 protein does not bind to murine STAT2 (**Figure 1**) (66). Hence, the inability of the NS5 protein to bind to murine STAT2 induces IFN to greatly limit DENV replication in mice (66). Such studies highlight the importance of NS5 as a virulence factor and a target for constructing live attenuated flavivirus vaccines.

### 4.5 Paramyxoviridae

#### 4.5.1 Bovine respiratory syncytial virus

Paramyxoviruses constitute a group of negative-sense, single-stranded, non-segmented RNA viruses. Bovine respiratory syncytial virus (BRSV) is classified in the Orthopneumovirus genus of the Paramyxoviridae family. BRSV is a significant cause of respiratory disease in cattle and a major contributor to the bovine respiratory disease (BRD) complex (156). Cattle vaccinated with the formalin-inactivated virus show enhanced severity when infected post-vaccination, suggesting the need for alternative BRSV vaccines (157). The genome of RSV codes for ten genes. NS1 and NS2 proteins have been identified as viral antagonists for the IFN system of hosts (67–70) (**Figure 1**). Various strategies using reverse genetics to remove viral IFN antagonists are being considered to generate attenuated BRSV vaccines (71). BRSV was engineered to delete the NS1 or NS2 gene, and the NS1 or NS2 gene-lacking BRSV induced higher levels IFNs than wild-type BRSV in bovine nasal fibroblasts and bronchoalveolar macrophages of immunized cattle (157). Furthermore, the recombinant BRSV was attenuated in IFN-competent cells *in vitro* and in calves, demonstrating that the NS1 and NS2 proteins are the critical determinants for the virulence of BRSV virulence. Recombinant BRSV lacking either NS1 or NS2 also induced higher BRSV-specific antibody titers in calves and greater priming of BRSV-specific, IFN-gamma-producing CD4(+) T cells for the protection against challenges with virulent BRSV (157). This finding delivers a prospect for developing a live attenuated BRSV vaccine.

The IFN antagonism has also been studied for human RSV as well. Recombinant human RSV with the NS2 deletion was highly attenuated in the lower respiratory tract in chimpanzees and induced significant resistance to challenges with wild-type RSV (71, 72) (**Figure 1**). These findings demonstrate that the deletion of an IFN antagonist from RSV is clinically attenuated and may provide increased protective immunity. However, controversial findings were reported in African green monkeys after evaluation of a series of recombinant human RSV. RSV with the NS2 deletion was not attenuated, whereas RSV with a double deletion of both NS1 and NS2 was over-attenuated and did not provide sufficient protection against wild-type RSV challenge (158). However, recombinant RSV with a double-deletion of M2-2 and NS2 exhibited attenuation and protection in monkeys. Despite the conflicting data, the results implicate the potential of IFN-deficient RSV as a live attenuated vaccine candidate (158, 159). The vaccine potential of IFN-suppression-deficient RSVs was further supported by the assessment in children (160–162). RSV/ΔNS2/Δ1313/I1314L contains three attenuating elements: deletion of the NS2 gene, deletion of codon 1313 in the RSV polymerase gene, and stabilizing missense mutation of I1314L in the polymerase. This triple mutant RSV was evaluated in RSV-seronegative children and shown to be restricted in replication but immunogenic and primed for potent antibody responses after natural exposure to wild-type RSV (161, 162). Taken together, the deletion of the NS2 gene leads to attenuation and immunogenicity of RSV. It validates the strategy to develop live attenuated vaccines by deleting the IFN-modulating function from RSV using reverse genetics.

#### 4.5.2 Measles virus

The measles virus (MeV) in the *Morbillivirus* genus is a highly immunotropic pathogen that can cause significant childhood morbidity and mortality worldwide. MeV infection induces immunosuppression in the host contributing to secondary infections and mortality (163). The MeV P gene codes for three proteins by mRNA editing; P as an essential polymerase cofactor, and V and C that function as viral antagonists of the IFN pathways (164, 165). The V protein is a multi-functional protein inhibiting IFN responses (**Figure 1**). The V protein interferes with IFN signaling by blocking STAT1/STAT2 nuclear translocation (53–56). In addition, it inactivates STAT1 and Tyk2 phosphorylation (57) and blocks the JAK1 function for IFN suppression (58, 59). The V protein also interacts with MDA5 and the RIG-I/TRIM25 regulatory complex in the IFN production pathway and inhibits downstream signaling (166–168). Besides the V protein, the C protein has also been determined to interfere with IFN transcription.

Furthermore, the nuclear localization signal and efficient nuclear accumulation are critical for the C protein to downregulate IFN-β production (**Figure 1**). Compared to the wild-type virus, a mutation in the nuclear localization signal of the C protein is a marker common to all vaccine strains of MeV (169). Amino acids essential for preventing STAT1 nuclear translocation were examined in the V and P proteins by screening the sequence of recombinant virus that could not antagonize STAT1 function, and three residues were identified in the shared domain of the P and V proteins; Y110, V112, and H115, with the Y110 being the most critical residue (164). A mutant virus was generated to harbor the three mutations and was used to assess the virulence in rhesus monkeys (170, 171). The inoculated monkeys showed a short duration of viremia and the absence of skin rash and other clinical signs observed with wild-type virus. This triple-amino acids mutant virus controlled the inflammatory response less efficiently, as measured by enhanced transcription of interleukin-6 and TNF-α in peripheral blood mononuclear cells from infected hosts. However, neutralizing antibody titers and virus-specific T-cell responses were equivalent in animals infected with either virus (172). These findings indicate that efficient MeV interactions with STAT1 are required to sustain virulence in a natural host by controlling the inflammatory response against the virus. Overall, these findings suggest that IFN-suppression defective MeV may have the potential as the vaccine for immunocompromised individuals.

#### 4.5.3 Nipah virus

Nipah virus is an emerging zoonotic pathogen, causing encephalitis and respiratory illness in humans and pigs. It belongs to the *Henipavirus* genus of the family *Paramyxoviridae*. Similar to MeV, the P gene of the Nipah virus codes for four proteins by mRNA editing; phosphoprotein P and three accessory proteins W, V, and C (173). These proteins possess a distinct IFN-antagonist activity, including the W protein acting as the inhibitor of the TLR-3 pathway, whereas proteins V and C function on the IFN signaling by interacting with STAT1/STAT2 (102, 174–177). However, the recombinant Nipah virus lacking the V, C or W protein still suppressed the IFN response as with the wild-type virus, indicating that the lack of each accessory protein does not significantly affect the inhibition of IFN signaling (174). Ferret challenge studies using a recombinant Nipha virus with deletion of the STAT1-binding motif also demonstrated the minor role of P, V, and W proteins in inhibiting IFN signaling (178, 179).

### 4.6 Orthomyxoviridae

#### 4.6.1 Human influenza virus

Influenza viruses belong to the family *Orthomyxoviridae* and contain single-stranded, negative-sense, eight segmented RNAs as the genome. Of seven genera of the family, influenza A and B viruses are of concern since they are frequently associated with respiratory disease in humans and animals. Two main subtypes are circulating in human population; H1N1 and H3N2. Of eight segments of the genomic RNA, segment 8 expresses two distinct proteins, nonstructural protein 1 (NS1) and nuclear export protein (NEP; also called NS2), using different reading frames from the same RNA segment. While NEP mediates the nuclear export of virion RNAs by acting as an adaptor between viral RNP complexes and the nuclear export machinery of the cell, NS1 is the viral antagonist for the IFN response of hosts (76, 180) (**Figure 1**). Influenza virus NS1 protein directly inhibits the production of IFNs by targeting RIG-I (180). In addition, the NS1 protein upregulates the inhibitors of JAK-STAT signaling and the suppressors of cytokine signaling (SOCS) family 1 and SOCS family 3 (76, 181), resulting in the downregulation of antiviral protein expression. NS1 protein also indirectly inhibits the IFN signal pathway and interacts with ISGs to antagonize the host’s antiviral response (180). Based on such information, influenza viruses were engineered to modify the NS1 functions. The NS1-modified viruses appeared clinically attenuated and retained the immunogenicity in various species of animals, including mice (182–185), pigs (186–188), horses (189, 190), poultry (191–194), macaques (195) as well as in humans (196–198). Influenza A virus containing a partial deletion in the NS1 gene provides solid evidence for clinical attenuation in animals while providing protective immunity against virulent challenges with the wild-type virus (199). Influenza virus A/WSN/33 (H1N1) expressing the mutant NS1 R38A/K41A showed a robust reduction of viral titers in the lungs of mice but triggered high levels of IFN-α/β production in the lung tissues (200). In addition, the NS1 R38A/K41A NS1 mutant virus induced high titers of neutralizing antibodies against heterologous influenza A virus and provided 100% protection in a mouse model against wild-type virus (200). Immunization of mice with the H1N1 influenza virus containing a shortened NS1 gene also showed the enhancement of influenza-specific CD8+ T-cellular response in the lungs and the reduction of proinflammatory cytokines with a lower extent of leukocyte infiltration after heterologous challenges, indicating that NS1-truncated influenza virus modifies not only effector T-cells but also specific immunoregulatory mechanisms (185). Influenza B viruses with NS1 mutations were also attenuated in animals while inducing adequate protection against both homologous and heterologous subtype challenges with influenza B viruses in BALB/c and C57BL/6 PKR(-/-) mice (201). Furthermore, the influenza B viruses with a truncated NS1 gene induced comparable cellular and humoral immune responses in both aged and young mice (202). It should be noted that most inbred mouse strains have deletions or point mutations in ISG Mx1 (203) which is an important antiviral factor in influenza virus infection. Therefore, using mice as a model to study IFN-influenza virus interaction should be of concern. Taken all together, these data demonstrated that the IFN-deficient system is applicable for manufacturing the IFN-sensitive influenza vaccine viruses.

#### 4.6.2 Swine influenza virus

Similar studies have been conducted for the swine influenza virus (SIV). Swine influenza is caused by the type A virus and regularly causes outbreaks in pigs. Swine influenza can cause high levels of illness in pig herds, but the mortality is low. Influenza viruses that circulate in swine are very different from influenza viruses circulating in people. Swine influenza viruses change genetically constantly. Pigs can be infected by avian influenza, human influenza, and swine influenza viruses. During the coinfection of pigs with influenza viruses from different species, the viruses can reassort, and new viruses can emerge as a mix of swine, human, and avian influenza viruses. Three main subtypes of influenza A virus have been isolated in pigs in the U.S.; H1N1, H3N2, and H1N2. Inactivated vaccines are less effective in protecting pigs, and thus, live-attenuated vaccines have been licensed and available in the U.S (204).. The SIV strain A/Swine/Texas/4199-2/98 (TX/98) H3N2 was used to investigate the role of NS1 protein for virulence, and the swine influenza virus NS1 mutants were shown to be attenuated in pigs (186, 187). Immunization of pigs with SIV NS1 mutant *via* the intranasal route provided complete protection against homologous and antigenically variant heterologous challenges (205, 206). Moreover, H3N2 NS1 mutant SIV-inoculated pigs displayed attenuated clinical symptoms and reduced viral titers despite a minor reduction in lung lesions when challenged with H1N1 heterosubtype SIV (205). A chimeric virus between the bat influenza virus and SIV was constructed to express a truncated NS1 protein. This virus induced remarkably higher levels of mucosal IgA response and antigen-specific IFN-γ secreting cells against the challenge virus in the lungs of immunized pigs (207).

#### 4.6.3 Avian influenza virus

The protective role of type I IFNs has also been studied for avian influenza virus in chickens. Avian influenza viruses continually circulate among wild birds and poultry worldwide, and the control of emerging influenza viruses for pandemic threats requires broadly protective vaccines. Influenza viruses with truncations in NS1 protein have shown broad-spectrum protection in birds and mammals, which has been correlated with the elevated IFN responses in vaccinated animals (208, 209). Immunologically improved strains of the avian influenza virus were identified by screening the subpopulation of viral vaccines (194). These strains appeared to have a small deletion in the NS1 protein or a single amino acid substitution in the polymerase 2 (PB2). These naturally occurring mutant viruses exhibited enhanced IFN-inducing phenotypes and protective immunity in chickens (194). Compared to an inactivated avian influenza vaccine, live-replicating NS1 mutant-avian influenza virus induced a more solid innate and highly cross-reactive serum antibody responses in immunologically immature chicken (210). Another study using H5N3 NS1 mutant virus also displayed similar levels of protection higher induction of IFN-β (211). However, the mutant virus reverted to wild-type phenotype within five back-passages in chickens, raising a concern about the stability of the NS1 mutant avian influenza vaccine (211). Taken all together, influenza viruses containing a truncated NS1 gene can boost a higher level of IFNs and adaptive immunity and confers protection from heterologous challenges in different species of animals. Such findings demonstrate that IFN-suppression negative mutant virus can be reprogramed to divert to an alternative vaccine candidate for veterinary diseases.

### 4.7 Coronaviridae

#### 4.7.1 Porcine epidemic diarrhea virus

Coronaviruses (CoVs) infect humans and animals, causing a variety of diseases with varying severity. Emerging and reemerging CoVs include porcine epidemic diarrhea virus (PEDV), porcine delta-coronavirus (PDCoV), Middle East respiratory syndrome coronavirus (MERS-CoV), swine acute diarrhea syndrome coronavirus, canine-like alphacoronavirus, SARS-CoV-1, and SARS-CoV-2 (212, 213). CoVs are divided into four genera; Alpha-CoV, Beta-CoV, Gamma-CoV, and Delta-CoV. Among these, only alpha-CoVs and beta-CoVs harbor nonstructural protein 1 (nsp1), which inhibits antiviral host responses. The IFN antagonistic function has been removed from the Alpha-CoV transmissible gastroenteritis virus (TGEV) and PEDV and examined for the effect of IFN suppression on viral pathogenesis in pigs. A recombinant TGEV was constructed to alter the specific C-terminal motif of nsp1, based on the crystal structure. The nsp1 mutation did not affect viral replication in cells but significantly reduced clinical outcome and pathogenicity in pigs (214). PEDV is also a significant veterinary pathogen in swine that requires a better vaccine for control. PEDV infection results in enormous economic losses to the pork industry worldwide and has emerged in the U.S. in 2014. Multiple proteins of PEDV have been shown to inhibit IFN responses during infection (214–219). The nsp1 protein of PEDV is the most potent viral IFN antagonist (214), and three residues of F44, N93, and N95 of nsp1 are critical for both type I and type III IFN suppression (52, 220). PEDV nsp1 suppressed both types of IFN responses by interrupting the IRF3 and CREB-binding protein association and suppressing transcription factors (52) (**Figure 1**). A replication-competent PEDV mutant with an IFN inactive version of nsp1 induced IFN response in IFN-responsive cells (52). It was further demonstrated that PEDV carrying the nsp1 N93/95A mutation triggered a significantly higher level of IFN response and induced 100% protection from severe diarrhea and death in neonatal piglets post-challenge (220). These findings suggest that nsp1 is an essential virulence determinant for CoVs, providing a potential paradigm for the development of a new vaccine based on IFN modification.

Nonstructural protein 15 (nsp15) of PEDV is the viral endoribonuclease (EndoU) and has an additional function as the IFN antagonist. For PEDV nsp15, three residues of H226, H241, and K282 were identified as critical amino acids for endoribonuclease activity. PEDV nsp15 can directly degrade the RNA levels of TBK1 and IRF3 and suppress the production of IFNs and ISGs, demonstrating that PEDV antagonizes host’s innate response to facilitate its replication (**Figure 1**). The endoribonuclease activity was removed from PEDV. The nsp15-modified PEDV reduced IFN antagonism with enhanced production of both type I and type III IFNs in cells and was clinically attenuated in infected piglets (216).

Nonstructural protein 16 (nsp16) of CoVs is 2’-*O*-methyltransferase (2’-*O*-MTase), and the nsp16 protein of PEDV also contains the methyltransferase. The methyltransferase activity was removed from nsp16 of PEDV. The nsp16 mutant PEDV increased type I IFN production in pigs and conferred complete protection following virulent PEDV challenge (221). Furthermore, infection with the inactive version of nsp1, nsp15, and nsp16 induced total and neutralizing antibody responses, and upon challenge with wild-type PEDV, no detectable clinical symptoms were observed in pigs (52). The pathogenic significance of 2’-*O*-MTase was also studied for SARS-CoV-1 and MERS-CoV. Both viruses with the nsp16 mutation resulted in IFN-based virulence attenuation and conferred the protection of mice from parental virus challenges (222, 223).

#### 4.7.2 Mouse hepatitis virus

Among animal CoVs, mouse hepatitis virus (MHV) has extensively been investigated (212). MHV is a beta-CoV and has been a research model to understand CoV genome replication, transcription, and pathogenesis. Studies showed that removal of IFN antagonism yielded stronger immunity to MHV. The nsp14 protein of MHV is a multifunctional protein with the N7-methyltransferase (N7-MTase) activity and is highly conserved among different CoVs. A N7-MTase-deficient recombinant MHV was constructed by replacing aspartic acid at position 330 and tyrosine at position 414 of nsp14, each with alanine (224). The N7-MTase-deficient MHV was highly attenuated in mice and showed delayed IFN production *in vivo*. Furthermore, this nsp14 mutant MHV induced an improved and long-term humoral immune responses and conferred complete protection against a lethal-dose of MHV (224). This study demonstrates the potential application of IFN antagonism to the design of live attenuated vaccines against CoVs circulating in humans and animals.

### 4.8 Arteriviridae

#### 4.8.1 Porcine reproductive and respiratory syndrome virus

Arteriviruses in the order *Nidovirales* infect a diverse species of mammals, including horses, pigs, possums, primates, and rodents (225). Arteriviruses have positive-sense, single-stranded RNA genomes and produce enveloped spherical particles. Porcine reproductive and respiratory syndrome virus (PRRSV) has evolved to carry various strategies for type I IFN antagonism and to evade host immune response. In PRRSV-infected pigs, the IFN response is meager and remains low in the lungs where the virus actively replicates (226, 227). In controlling PRRSV infection in pigs, viral suppression of innate immunity, delayed response of the adaptive immunity, and antigenic heterogeneity of PRRSV are the keys (228). Studies attempted to express various types of IFNs using PRRSV as a vector. IFN-expressing PRRSV increased the IFN levels in pigs after immunization and enhanced the protection against the secondary PRRSV challenge (127, 128). The porcine IFN-α-expressing plasmid as an adjuvant for PRRSV vaccines also induced a prolonged adaptive immune response in pigs (129). Instead of exogenous administration of IFNs, endogenous expression of IFNs seems to be a better way to enhance adaptive immunity. Indeed, a PRRSV strain of IFN-inducing phenotype PRRSV has been shown to improve the neutralizing antibody production (229).

The nsp1β protein of PRRSV is a potent IFN antagonist inhibiting both types I IFN production and downstream signaling (60–63) (**Figure 1**). Mutant PRRSVs were generated to remove IFN-suppression function from nsp1β, and pigs infected with nsp1β-mutant PRRSVs induced higher levels of IFN-α and ISGs (230, 231). The NK cell function and the IFN-γ level were increased in the lungs of pigs infected with the nsp1β mutant virus (230). Moreover, pigs inoculated with a nsp1β-mutant PRRSV exhibited shorter duration and lower titers of viremia, and a more robust PRRSV-specific antibody response. The neutralizing antibody titers were also higher than those of control pigs, indicating that the IFN-suppression-negative PRRSV mutants are clinically attenuated (231). These studies demonstrate the role of type I IFNs in priming the adaptive immune response in pigs and provide evidence that IFN antagonism-deficient PRRSV may be developed as a new vaccine candidate. Removing IFN antagonism from the virus is a reasonable strategy for developing next-generation vaccines.

#### 4.8.2 Equine arteritis virus

Evidence is available for another arterivirus, the equine arteritis virus (EAV), for the removal of type I IFNs in developing adaptive immunity and viral clearance. The nsp2 protein of EAV contains DUB activity and is responsible for the viral suppression of innate immunity (232). Thus, a DUB-negative mutant EAV was constructed. Vaccination of horses with the DUB-negative mutant virus induced higher levels of IFNs and adaptive immune responses (233). Taken together, the removal of IFN antagonism is an important strategy for developing novel vaccines for arteriviruses.

## 5 Viral antagonist for NF-κB and as a potential target for attenuated vaccine development

NF-κB is a transcription factor and functions as a hub of complex signaling networks in the cell. NF-κB signaling contributes to immunity, inflammation, cell growth, development, cancer, and other cellular processes. The NF-κB pathway links pathogen and cellular signals and organizes cell resistance to invading pathogens. Many viruses have developed distinct mechanisms to suppress or activate the NF-κB signaling pathway to promote virus replication or cell proliferation (234). Virus-induced NF-κB activation is linked to overproduction and uncontrolled release of proinflammatory markers resulting in a “cytokine storm”. SARS-CoV-2 activates the NF-κB signaling pathway and causes cytokine storm-like acute respiratory distress syndrome (235, 236). Targeting the NF-κB pathway has been considered a potential treatment for COVID-19 patients (236). The influenza virus can also activate NF-κB signaling and induces cytokine storm-like symptoms in infected hosts (237). Activation of NF-κB and production of proinflammatory cytokines are often synergistic during coinfection. Studies using PRRSV in pigs show that the virus can activate NF-κB signaling and elevate proinflammatory cytokine production when coinfected with Streptococcus suis. The cytokine storm-like production of inflammatory cytokines results in more severe clinical outcomes in coinfected animals (238). Thus, viral activation of NF-κB signaling and a cytokine storm-like event are additional concerns for attenuated viral vaccines. A study showed that the reduction of systemic inflammation and a boost of protective responses when combining an NF-κB inhibitor as an adjuvant with a vaccine in the influenza mice challenge model (239). Since the NF-κB signaling contributes to numerous cellular processes, potential off-target effects by NF-κB inhibition may be actuated and cause a systematically undesirable consequence. Thus, further research is required for developing NF-κB-activation-negative viruses as feasible live-attenuated vaccines. Nevertheless, by the fine-tuning removal of the NF-κB activation, a newly generated virus is anticipated to relieve the clinical severity attributed to cytokine upregulations. By reprogramming the NF-κB activation, the newly generated virus is expected to relieve the clinical severity attributed to cytokine upregulation.

This concept is useful for pigs with PRRSV as a new approach to vaccine developments. For activation of NF-κB, the nucleocapsid (N) protein is the sole viral protein for PRRSV (240, 241). Studies show that the nuclear localization signal (NLS) in N is the essential domain for binding to PIAS1. PIAS1 is the negative regulator for NF-κB, so the binding of viral N protein to PIAS1 results in the release of p65 from PIAS1 and renders NF-κB activation (241). The NLS-modified N protein loses the ability for NF-κB activation and induces significantly lower levels of NF-κB-mediated inflammatory cytokines in cells (241). Studies using the NLS-null PRRSV infection show milder clinical signs and a shorter duration of viremia with higher titers of antibodies in infected pigs, demonstrating the association between the NLS motif of N protein and clinical attenuation of PRRSV infection (242–244). These studies provide evidence that NF-κB-activation-negative virus can induce milder clinical symptoms and higher humoral immune response than wild-type virus during coinfection of pigs. NF-κB activation function can be eliminated from RNA viruses using reverse genetics tools, and clinically attenuated vaccine candidates can be developed.

## 6 Conclusion

Numerous mechanisms for how viruses fight against the host immune system have been identified. Since type I IFNs are critical components of the host innate immunity and for the development and maturation of adaptive immunity, many attempts have been made to remove IFN suppression functions from different viruses. Such approaches may enhance the IFN response upon immunization. With the help of recent advances in molecular virology and reverse genetics technology, it is possible to construct mutant viruses of which phenotypes are IFN-suppression-deficient. Subsequently, IFN-suppression-negative viruses have shown to induce better immune responses and confer partial to complete protections from both homologous and heterologous challenges in the respective animal species, such as mice, pigs, chickens, and cattle (**Table 1**). Still remaining challenges include:

1) Identification of IFN-suppressive genes and active sites from target viruses: It requires an in-depth understanding of the structure-function relationships of viral proteins and their role in the IFN signaling cascade.
2) Multigenic nature of viral IFN antagonists and introduction of multiple mutations: Viruses have evolved to equip with multiple proteins for IFN suppression such that their antagonism can be compensated even when one protein fails by evolutionary mutations. The strategy to overcome the multigenic nature of viral IFN antagonism is to introduce mutations to the most potent antagonist or simultaneous mutations to several antagonists.
3) Lethality of mutations for viral infectivity and viability: Many RNA viruses carry only a limited number of genes essential for replication and survival in a host. Deletion of a functional gene is often lethal for infectivity, and accurate measurements of the gene-wide mutations may be needed for the possible substitutions of amino acids and the removal of IFN antagonism.
4) Reversion of mutant viruses to virulence: RNA-dependent RNA polymerase lacks a proof-reading activity with an exception of coronaviruses that are found to contain an exonuclease as a proofreading enzyme. As a result, RNA viruses are prone to high rates of genetic mutations which may allow the reversion of IFN-negative attenuated viral mutants back to virulence. Deletion of the functional domain or amino acids instead of point mutations has been successful for some viruses to block their reversion to wild-type.
5) Over-attenuation of mutant viruses: Deletions or substitutions in a functional domain may confer over-attenuation of virus mutants. Continuous passages in cell culture have been attempted to increase viral titers. Cell culture passages in established lines may not provide immunological pressure but can allow viral adaptation for efficient replication and production of high viral titers.

**Table 1:** The reprogramming of mutant viruses that their IFN evasion functions were eliminated by reverse genetics and the protective efficacies in natural host animal species following vaccination and virulent challenges.

Virus	Affected Animal Species	Disease and Risk	Viral IFN Antagonist	IFN Suppression Mechanism	Animal Vaccination	Protective Efficacy
FMDV	Cloven-hoofed animals	Blisters	L^pro^	IRF3 ↓ ISG15 ↓	Mice (142) Pigs (143, 144) Cattle (145)	100% in pigs 100% in cattle
Sindbis virus	Mosquito, Avian	Rash-arthritis syndrome	Nsp1	JAK-STAT signaling ↓	Mice (147–149)	N/A
WNV	Mosquito, Avian	Encephalitis, Meningitis	NS5	STAT1 phosphorylation ↓	Mice (155)	N/A
BRSV	Bovine	Minimal to extreme respiratory diseases	NS1, NS2	JAK-STAT signaling ↓	Cattle (157)	↑ in cattle
RSV	Human, Primate	Bronchitis, Pneumonia	NS2	JAK-STAT signaling ↓	Chimpanzees (71, 72), Monkeys (158, 159)	↑ in chimpanzees No in monkeys
Measles virus	Human	Acute respiratory, Systemic illness	V	MDA5 and RIG-I/TRIM25 ↓ STATs nuclear translocation ↓ STAT1phosphorylation ↓ JAK1 function ↓	Rhesus monkeys (170, 171)	N/A
			C	IFN transcription ↓		
Nipah virus	Fruit Bat, Human	Acute respiratory, Encephalitis	W	TLR-3 pathway ↓	Ferrets (178, 179)	N/A
			V and C	STAT1/STAT2 ↓		
Influenza virus	Mammal, Avian	Respiratory	NS1	Targeting RIG-1 ↓ SOCS family ↑	Mice (182–185) Pigs (186–188, 205–207) Horses (189, 190) Poultry (191–194, 210) Macaques (195)	100% in pigs ↑ in pigs ↑ in poultry
PEDV	Swine	Enteritis in neonates	nsp1	IRF3 and CREB-binding protein ↓	Pigs (216, 220, 221)	100% in pigs
			nsp15	IRF3 ↓		
			nsp16	RIG-I and MDA5 ↓		
MHV	Murine	Enteritis, Neurologic, Hepatitis	nsp14	N/A	Mice (224)	100% in mice
PRRSV	Swine	Reproductive, Respiratory	nsp1β	ISGF3 nuclear translocation ↓ STAT1 phosphorylation ↓	Pigs (231)	↑ in pigs
EAV	Equine	Abortion, Respiratory	nsp2	ISG15 ↓	Horses (233)	↑ in horses

FMDV, foot-and-mouth disease virus; WNV, West Nile virus; BRSV, bovine respiratory syncytial virus; RSV, respiratory syncytial virus; PEDV, porcine epidemic diarrhea virus; MHV, mouse hepatitis virus; PRRSV, porcine reproductive and respiratory syndrome virus; EAV, equine arteritis virus. ↑ (up arrow) indicates "enhanced protection". ↓ (down arrow) indicates "down-regulation". N/A indicates "Not available".

The combination of research and new ideas will be vital in successfully developing future vaccines for veterinary diseases.

## Author contributions

C-MS and DY contributed to developing concepts and design of the review article. C-MS performed the literature search, retrieved relevant articles, and prepared a draft of the original manuscript. C-MS and DY wrote the manuscript. YD, RR, and QW provided critical comments and revised the manuscript. All authors contributed to the article and approved the submitted version.
